# Supplementary material for: Combined and Sequential Treatment with Deep Brain Stimulation and Continuous Intrajejunal Levodopa Infusion for Parkinson’s Disease
Source: J Pers Med. 2021 Jun 12;11(6):547. doi: 10.3390/jpm11060547 (PMC8231578; doi:10.3390/jpm11060547)
Supplement: Supplementary file 1 [file jpm-11-00547-s001.zip › Supplemental Table S1 - Search Criteria.pdf]

Supplemental Table S1: Search Criteria

Embase		
#	**Searches**	**Results**
1	exp Parkinson disease/ or PARKINSON*.ti,ab,kw,hw.	228495
2	(exp levodopa/ or exp carbidopa plus entacapone plus levodopa/ or exp carbidopa plus levodopa/ or exp levodopa methyl ester/ or (duodopa or levodopa or (levodopa adj2 carbidopa) or l-dopa).ti,ab,kw,rn,hw.) and (exp Intestine, Small/ or (infusion or intrajejunal or jejenum or continuous*).ti,ab,kw,hw.)	3608
3	brain depth stimulation/	44674
4	("Deep brain stimulation" or dbs or "Subthalamic nucleus stimulation" or (STN adj2 stimulation) or ((gpi or globus) adj 2 stimulation) or (Thalamic adj2 stimulation)).ti,ab.	27127
5	3 or 4	53445
6	1 and 2 and 5	567
Medline		
#	**Searches**	**Results**
1	exp Deep Brain Stimulation/	9167
2	Deep brain stimulation.ti,ab.	11612
3	dbs.ti,ab.	10795
4	Subthalamic nucleus stimulation.ti,ab.	428
5	((gpi or globus) adj 2 stimulation).ti,ab.	0
6	(Thalamic adj2 stimulation).ti,ab.	700
7	(STN adj2 stimulation).ti,ab.	924
8	1 or 2 or 3 or 4 or 5 or 6 or 7	17871
9	exp Parkinson Disease/	69221
10	parkinson*.ti,ab,kf.	124970
11	9 or 10	132529
12	(exp Levodopa/ or (duodopa or levodopa or (levodopa adj2 carbidopa) or l-dopa).ti,ab,kw,rn.) and (exp Intestine, Small/ or (infusion or intrajejunal or jejenum or continuous*).ti,ab,kf.)	1610
13	8 and 11 and 12	184
