# Supplementary material for: Combined and Sequential Treatment with Deep Brain Stimulation and Continuous Intrajejunal Levodopa Infusion for Parkinson’s Disease
Source: J Pers Med. 2021 Jun 12;11(6):547. doi: 10.3390/jpm11060547 (PMC8231578; doi:10.3390/jpm11060547)
Supplement: Supplementary file 1 [file jpm-11-00547-s001.zip › Supplemental Table S2 - Comparing response to second advanced therapy.pdf]

Supplement 2. **Table S2.** Comparing response to second advanced therapy.

	2nd treatment beneficial (n=12)	2nd treatment not beneficial (n=5)	p-value
Initial treatment	CLI 7 DBS 5	CLI 2 DBS 3	0.800
Sex F (n out of total n; (%))	3/12 (25%)	2/5 (40%)	0.787
Mean age at PD diagnosis in years (SD)	46.3 (6.4)	43.6 (7.8)	0.442
Mean age at initiation initial treatment in years (SD)	56.4 (7.8)	51.2 (7.2)	0.279
Mean initial effect of first treatment, 0-4 (SD)	2.6 (1.0)	3.2 (0.5)	0.279
Initial treatment beneficial	7	5	0.077
Mean interval between treatments in months (SD)	55.3 (24.7)	56.2 (27.2)	0.959
Mean final LEDD (SD, n)	1337 (862, n=11)	1733 (912 n=5)	0.441

CLI: Continuous Levodopa/carbidopa Infusion, DBS: Deep Brain Stimulation, LEDD: levodopa equivalent daily dose, PD: Parkinson's disease, SD: standard deviation.
